# COVID-19 Infection Risk Following Elective Arthroplasty and Surgical Complications in COVID-19-vaccinated Patients: A Multicenter Comparative Cohort Study

**DOI:** 10.1016/j.artd.2022.09.005

**Published:** 2022-09-27

**Authors:** Seyed Peyman Mirghaderi, Maryam Salimi, Alireza Moharrami, Reza Hosseini-Dolama, Seyed Reza Mirghaderi, Milad Ghaderi, Mehdi Motififard, Seyed Mohammad Javad Mortazavi

**Affiliations:** a Joint Reconstruction Research Center, Tehran University of Medical Sciences, Tehran, Iran; b Students' Scientific Research Center (SSRC), Tehran University of Medical Sciences, Tehran, Iran; c Student Research Committee, School of Medicine, Isfahan University of Medical Sciences, Isfahan, Iran; d Department of Orthopedic Surgery, Kashani University Hospital, School of Medicine, Isfahan University of Medical Sciences, Isfahan, Iran

**Keywords:** Arthroplasty, COVID-19, Elective, Vaccines, Venous thrombosis

## Abstract

**Background:**

We aimed to determine symptomatic Coronavirus disease 2019 (COVID-19) rates within 1 month of elective arthroplasty for vaccinated individuals and to determine whether vaccination guarantees protection against COVID-19 after arthroplasty (primary outcome). In addition, the 90-day surgical complications were compared to those of an unvaccinated group (secondary outcome).

**Methods:**

A prospective cohort study was conducted on elective joint arthroplasty patients at 3 tertiary hospitals in 2 major cities (Tehran and Isfahan) in our country (Iran). The outcomes of the COVID-19-vaccinated group were assessed between October 2021 and March 2022. Ninety-day surgical complications were compared with a historical cohort of unvaccinated patients treated earlier in the pandemic (April 2020-March 2021).

**Results:**

The study included 1717 consecutive patients: 962 vaccinated and 755 unvaccinated. In the vaccinated group, 38 patients (3.9%) contracted COVID-19, 4 (10.5%) were hospitalized again, and none required intensive care unit admission. The multivariate logistic regression analysis revealed that COVID-19-positive cases are more likely to be female (odds ratio [OR] = 12.5), to have visitors to their home (OR = 4.7), and to stay longer in the hospital (OR = 1.2) than COVID-19-negative cases. Compared to unvaccinated patients, the postoperative COVID-19 rate was not significantly different (3.9% vs 2.4%, *P* = .07). The incidence of surgical complications was similar between the 2 groups (*P* > .05).

**Conclusions:**

The vaccination does not provide a guarantee that a patient will not contract COVID-19 following their arthroplasty surgery, especially in a region with a high rate of COVID-19. We believe reasonable perioperative COVID-19 precautions may be warranted even in vaccinated patients.

## Introduction

It has been a challenging year for many orthopedic surgeons worldwide due to the Coronavirus disease 2019 (COVID-19) which halted their professional life [1]. As a result of the COVID-19 pandemic, access to elective health care has been adversely affected, especially for orthopedic surgical procedures such as joint replacements [[1], [2], [3], [4], [5], [6], [7]]. A COVID-19 pandemic–related surgical delay exacerbated osteoarthritis, caused discomfort, and lowered quality of life in severe osteoarthritis patients [[8], [9], [10], [11], [12], [13]]. It is possible for surgical patients to acquire COVID-19 infections despite low rates of COVID-19 infection in the area [14,15]. As a result of a recent study by our team [15], 2.4% of unvaccinated patients (18 out of 755) who underwent an elective arthroplasty contracted COVID-19 within 30 days of discharge. In urgent cases, the rate may reach 3.3% [16]. Consequently, these patients are at a higher risk of postoperative pulmonary complications, thromboembolic events, and mortality [[17], [18], [19]].

There are several COVID-19 vaccines available worldwide that have demonstrated high effectiveness in preventing the infection and lowering COVID-19-related deaths [[20], [21], [22], [23], [24], [25], [26], [27]]. The preoperative vaccination could significantly reduce the risk of COVID-19 complications, thus enabling elective surgery to be performed safely [17,[28], [29], [30]]. In order to perform a safe surgical procedure, there must be a minimum amount of nosocomial transmission. The COVIDSurg Collaborative study [17] revealed that surgical patients had a more favorable number needed to treat to prevent 1 death associated with COVID-19 in 1 year. Thus, they recommended that surgical patients be given priority over the general population for vaccination. Furthermore, widespread vaccinations of surgical patients enabled safe resumption of elective surgery services [17]. On the other hand, COVID-19 variants of concern (VOCs) have raised questions regarding the effectiveness of vaccines as a preventative measure against infections, transmission, and ongoing pandemics [31]. The emergence of “vaccine breakthroughs” has been observed extensively among people who have been fully vaccinated with all types [31,32]. There are 4 Omicron sublineages classified as VOCs: BA.1, BA.2, BA.4, and BA.5 [33]. Recent discoveries of the sublineages BA.4 and BA.5 have raised several concerns, including greater transmissibility, greater resistance to vaccines, and an increased risk of reinfection [33]. In recent studies, researchers have discovered that these 2 sublineages, BA.4 and BA.5, are linked to an increased risk of reinfection following vaccination and exhibit distinct pathogenic characteristics [[34], [35], [36]]. Due to their ability to resist antibodies produced by vaccination and previous infection, they may cause a new wave of infection [34,35]. In addition to increased transmission, morbidity, and mortality, these VOCs impair diagnosis, cause reinfection in previously infected individuals, and lead to vaccine breakthroughs in fully vaccinated individuals [37].

Another challenge is the possible impact of COVID-19 vaccination on surgery complications, particularly venous thromboembolic (VTE) events [38]. There is a concern about the risk of VTE after COVID-19 vaccination. Although large-scale studies on vaccinated individuals showed no higher risk of thrombosis than those in the general population [39], few reports have noticed an unusual but devastating complication of VTE associated with thrombocytopenia observed after the administration of adenoviral vector-based vaccines (AstraZeneca and Johnson & Johnson) [39,40]. Additionally, some rare VTE events have been reported with m-RNA-based vaccines (Pfizer/BioNTech and Moderna) [[41], [42], [43]]. Regardless of the vaccine, joint replacement patients are at risk of developing VTE due to the duration of the procedure and reduced mobility during postoperative recovery. After the surgery, patients are treated with anticoagulants for up to 1 month to reduce their risk of developing VTE [44]. Variable rates of VTE at 3 months after a total hip arthroplasty (THA), total knee arthroplasty, and unicompartmental knee arthroplasty are reported (up to 5% for deep vein thrombosis) [45].

This study was designed in response to the lack of evidence regarding the nosocomial COVID-19 infection rate among vaccinated patients after elective joint replacement and thromboembolic issues afterward. The objectives of the current study are (1) to report the rate of symptomatic COVID-19 among vaccinated individuals within 1 month after discharge from elective hip and knee arthroplasty and (2) to evaluate the incidence of surgical complications, specifically VTE, at a 3-month follow-up after an arthroplasty surgery and compare it with the historical unvaccinated cohort.

## Material and methods

The study was approved by our institutional review board, and no ethical concerns were identified. Informed consent was obtained from all patients before participation in the study.

### Study design, setting, and participants

A prospective cohort study was carried out on adult patients who underwent an elective joint arthroplasty—including total knee arthroplasty, THA, and unicompartmental knee arthroplasty—at 3 tertiary hospitals in 2 large cities in our country (Iran): Imam Khomeini (Tehran, university hospital); Atieh (Tehran, private hospital); and Saadi hospitals (Isfahan, private hospital). In these 2 cities, Tehran and Isfahan, these centers are the top arthroplasty centers and admit a large number of patients. Two periods were chosen to compare vaccinated and unvaccinated patients regarding 90-day surgical complications: October 2021-March 2022 (second period), when the country population was vaccinated widespread and from April 2020 (start of the pandemic, first period) to March 2021. The study's primary outcome is contracting symptomatic COVID-19, confirmed by positive reverse transcription polymerase chain reaction (RT-PCR), computed tomography scan, or clinical evaluation by an infectious specialist. The secondary outcome is the surgical complications and VTE event by 90-day postoperative follow-up.

The exclusion criteria are (1) other types of arthroplasty; (2) nonelective arthroplasty, such as traumatic cases underwent THA; (3) not fully vaccinated (2 doses, second dose >2 weeks before surgery); (4) not willing to participate.

### Outcomes and variables

We collected data on the variables of interest from our arthroplasty registry, follow-up visits, and weekly phone calls:

1. Demographics and clinical data: age, sex, body mass index, Charlson Comorbidity Index.
2. Surgery information: type and indication of the surgery, revision or primary surgery, indication for revision, hospital type, hospitalization length for surgery, delay in surgery.
3. COVID-19-related information: previous infections of COVID-19, hospitalizations in the same room, having patients' companion 24 hours, having visitors at the hospital or at home after discharge, contracting COVID-19 within a month after discharge, the time between discharge and onset of symptoms, the hospital length of stay, intensive care unit (ICU) admission, the need for mechanical ventilation, and deaths caused by COVID-19. The COVID-19 weekly incidence rate in the country was retrieved from https://www.worldometers.info. Patients who had visitors to the home after discharge from hospital are theoretically at risk of catching COVID-19 infection if visitors are COVID-19-positive and asymptomatic at home after discharge. COVID incubation, defined as the period between exposure/inoculation and onset of symptoms, is about 6 days, but 10% of patients have an incubation period of over 14 days, so we followed up patients for 30 days after the surgery [46].
4. Vaccination status: number of doses administered and the brand of the vaccine that was documented on the participants’ vaccine card.
5. Arthroplasty-related complications, measured at 3-month follow-up: venous thromboembolic events (VTE), surgical site infection (SSI)/periprosthetic joint infection (PJI), wound complications (dehiscence/drainage), etc.

### Data collection

All patients were followed up for 30 days for COVID-19 and 90 days for surgical complications. During weekly phone calls, patients were asked about recent COVID-19 symptoms, including fever, myalgia, sore throat, headache, dyspnea, cough, and diarrhea. The RT-PCR test was performed on nasopharyngeal and oropharyngeal swabs of any patients with suspicious symptoms. Infectious disease specialists confirmed infections in suspicious patients. Data regarding their COVID-19 illness and disease course were recorded. The patients are routinely seen at our centers’ clinics 1 week, 6 weeks, 3 months, 6 months, and 12 months after the surgery. Surgical complications are recorded at the 3-month follow-up visit as a secondary outcome.

### Preoperative COVID-19 protocols

There was a COVID-19 ward at the university hospital investigated in the present study, while private centers did not admit people with this disease. All centers performed COVID-19 screening by taking the patient's medical history and a screening questionnaire, history of travel to another state, by evaluating symptoms, checking the body temperature, and monitoring their pulse oximetry. Afterward, patients were assessed by laboratory and imaging modalities to rule out COVID-19, which differed between cities. In Tehran, all patients were examined twice by RT-PCR before the surgery (48 hours before and on the day of the surgery). However, in Isfahan, 2-view chest x-rays were taken to detect airspace opacities or any suspicious findings. The patients who were not suspicious of COVID-19 were scheduled for their surgery. It is noteworthy to mention that all centers prescribe aspirin 325 mg twice daily for 6 weeks as VTE prophylaxis. Regardless of their COVID-19 history, all patients received the same VTE chemoprophylaxis.

### Statistical analysis

Data were gathered in the Excel 2016 software (Microsoft Corporation, Redmond, WA), and the SPSS version 23 (IBM, Armonk, NY) was used for data analysis. The Kolmogorov-Smirnov and Shapiro-Wilk tests were used to determine data normality. The chi-square and Fisher's exact tests were used to compare the qualitative variables across groups (COVID-19-positive and COVID-19-negative patients, vaccinated and nonvaccinated patients). The quantitative variables were compared using an independent sample T-test. Using the logistic regression test, we investigated the potential risk factors that were statistically significant in the univariate analysis. A significance level of *P* < .05 (2-sided) was considered.

## Results

**Figure 1:**
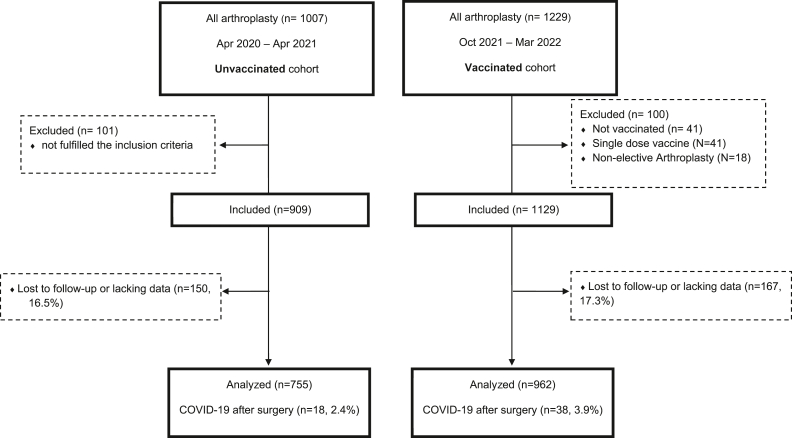
Patient enrollment flow diagram and rate of postoperative symptomatic COVID-19 within 1 month as the primary outcome of the study.

A total of 1717 consecutive patients were included in the analysis, 962 of them in the second period after COVID-19 vaccination (October 2021-March 2022) and 755 of them in the first period before COVID-19 vaccination (April 2020-March 2021). Figure 1 represents the patients' enrollment flow diagram.

**Figure 2:**
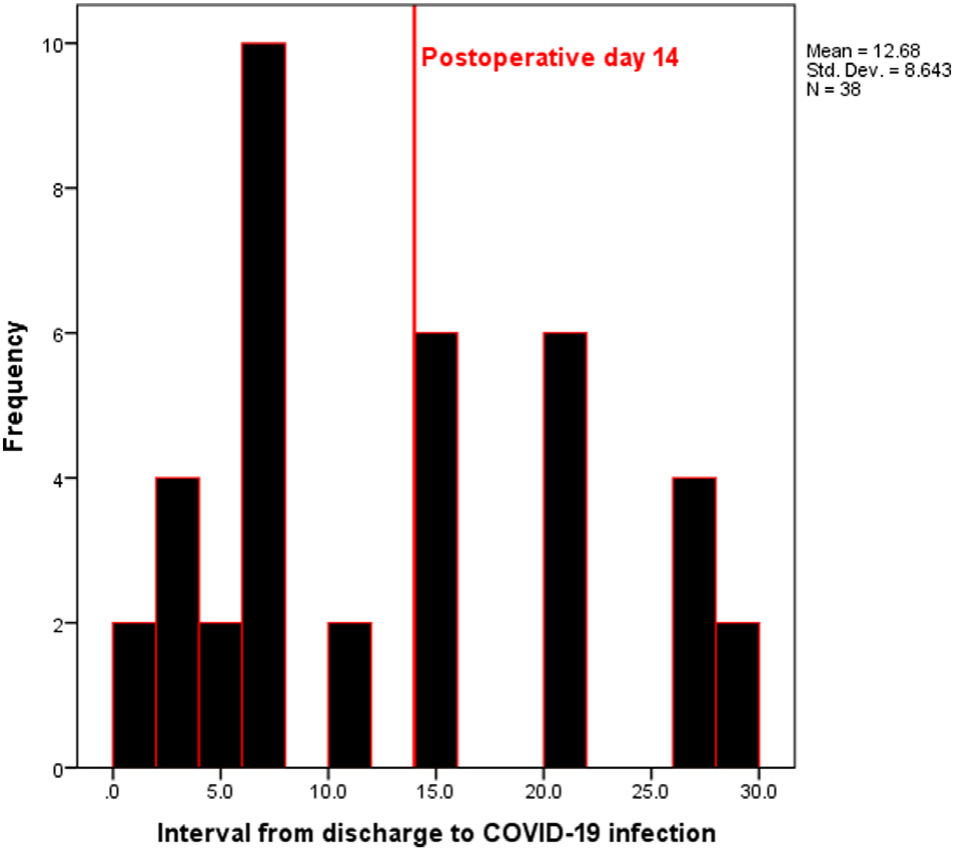
Day interval from discharge to COVID-19 infection.

**Figure 3:**
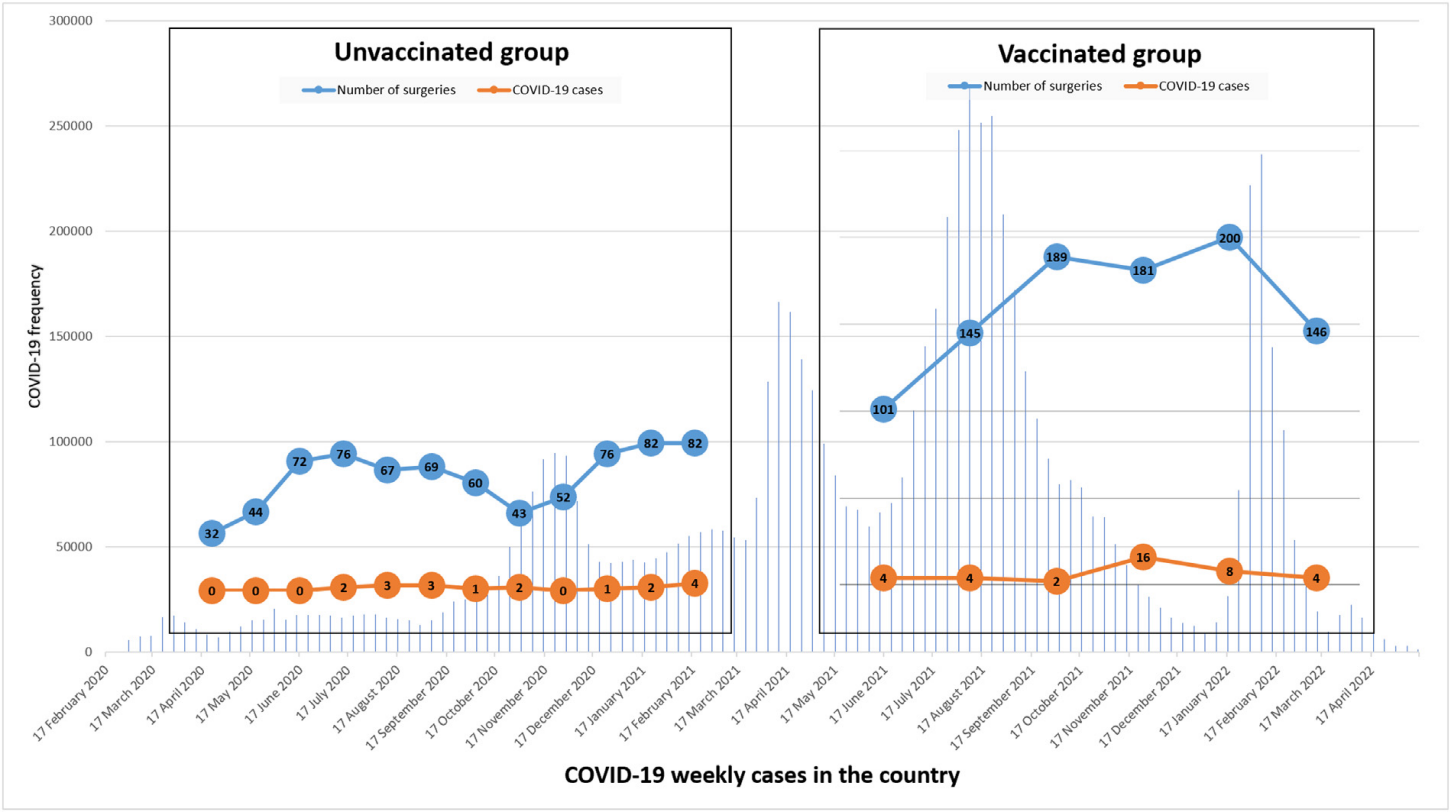
The trend of arthroplasty surgeries (blue), COVID-19-positive cases (orange) in the study, and the COVID-19 incidence in our country (histogram chart). Data of the COVID-19 incidence in our country is from Worldometers (https://www.worldometers.info/).

Table 1 shows the cohort of vaccinated patients and comparison of COVID-19-positive and COVID-19-negative patients. Among the vaccinated individuals, 38 (3.9%) developed COVID-19 within 1 month after the elective joint replacement surgery. There were 4 (10.5%) patients hospitalized for respiratory symptoms for a mean of 4 days, but none of them required ICU admission, and all recovered without sequalae. The patient's onset of COVID-19 symptoms began 12.7 ± 8.6 days after discharge (Fig. 2). The trend of arthroplasty surgeries and COVID-19-positive cases in our study and the COVID-19 incidence in our country are shown in Figure 3.

**Table 1:** Comparison of COVID-19-positive and COVID-19-negative vaccinated patients within 1 month after elective joint replacement.

	COVID-19 within 1 mo after arthroplasty (N = 962)		*P* value
	Positive (n = 38, 3.9%)	Negative (n = 924, 96.1%)	
Demographics			
Age (y)	63.4 ± 10.5	64.2 ± 11.2	.66
Female sex, n (%)	37 (97.4%)	714 (77.2%)	**.001**^a^
			**OR**^b^**(95% CI) = 12.5 (1.7-91.8)**
BMI (kg/m^2^)	27.5 ± 3.3	28.2 ± 4.4	.33
Charlson Comorbidity Index (CCI)	0.6 ± 1.0	0.7 ± 1.0	.84
City			.34
Tehran (434, 45.1%)	20 (52.6%)	414 (44.8%)	
Isfahan (528, 54.9%)	18 (47.4%)	510 (55.2%)	
Surgical information			
Joints, n (%)			.60
Hip (237, 24.6%)	8 (21.1%)	229 (24.8%)	
Knee (725, 75.4%)	30 (78.9%)	695 (75.2%)	
Surgery, n (%)			.11
Primary (930, 96.7%)	35 (92.1%)	895 (96.7%)	
Revision (32, 3.3%)	3 (7.9%)	29 (3.1%)	
Indication of the surgery, n (%)			.12
OA (834, 86.7%)	31 (81.6%)	803 (87.0%)	
AVN (68, 7.1%)	2 (5.3%)	66 (7.2%)	
RA (12, 1.2%)	0	12 (1.3%)	
DDH (12, 1.2%)	2 (5.3%)	10 (1.1%)	
Revision (32, 3.3%)	3 (10.5%)	29 (3.0%)	
Other (hemophilia, septic, tumor) (4, 0.4%)	0	4 (0.4%)	
Hospital type, n (%)			.27
Private (862, 89.6%)	32 (84.2%)	830 (89.8%)	
University (100, 10.4%)	6 (15.8%)	94 (10.2%)	
Hospital stay for surgery, n (%)	3.2 ± 1.3	2.8 ± 1.4	**.014**^a^
			**OR**^b^**(95% CI) = 1.2 (1.01-1.46)**
COVID-19-related information			
Delayed surgery due to COVID-19 (112, 11.6%), n (%)	6 (15.8%)	106 (11.5%)	.48
Delayed duration (mo)	6.0 ± 4.6	6.9 ± 6.1	.72
History of COVID-19 infection (286, 29.8%)	8 (21.1%)	278 (30.1%)	.26
Number of patients hospitalized in the same room	1.1 ± 0.8	1.4 ± 1.5	.22
Having a patient companion 24 h	25 (65.8%)	652 (70.7%)	.53
Having relatives’ visit at the hospital	14 (36.8%)	260 (28.2%)	.25
Having relatives’ visit at home	34 (89.5%)	616 (66.7%)	**.001**^a^
			**OR**^b^**(95% CI) = 4.7 (1.6-13.4)**
Vaccination information			
Number of doses, n (%)			.07
Two doses (591, 61.4%)	18 (47.4%)	573 (62.0%)	
Three doses (371, 38.6%)	20 (52.6%)	351 (38.0%)	
First and second doses (n = 962, 100.0%)			.15
Sputnik V (16, 1.7%)	1 (2.6%)	15 (1.6%)	
Oxford/AstraZeneca (200, 20.8%)	12 (31.6%)	188 (20.3%)	
Sinopharm (739, 76.8%)	24 (63.2%)	715 (77.4%)	
Pfizer-BioNTech (7, 0.7%)	1 (2.6%)	6 (0.6%)	
Third dose (n = 371, 38.6%)	20 (52.6%)	351 (38.0%)	.87
Sputnik V (8, 2.2%)	0	8 (2.2%)	
Oxford/AstraZeneca (89, 24.0%)	5 (25%)	84 (23.9%)	
Sinopharm (270, 72.8%)	15 (75%)	255 (68.7%)	
Pfizer-BioNTech (4, 1.1%)	0	4 (1.1%)	
Time of the last dose (weeks before surgery)	7.8 ± 3.7	7.3 ± 4.1	.51
Arthroplasty-related complications (3 mo), n (%)			
Venous thromboembolism (8, 0.8%)	0	8 (0.9%)	.56
Periprosthetic joint infection/surgical site infection (11, 1.1%)	1 (2.6%)	10 (1.1%)	.38
Wound complications (dehiscence/ persistent drainage) (61,6.3%)	4 (10.5%)	57 (6.2%)	.38
Other (periprosthetic fracture, dislocation, joint stiffness) (7, 0.7%)	1 (2.6%)	6 (0.6%)	.16
Revision surgery (0)	0	0	-
Mortality (1, 0.1%)	0	1 (0.1%)	-

BMI, body mass index; CI, confidence interval; OA, osteoarthritis; AVN, avascular necrosis; RA, rheumatoid arthritis; DDH, developmental dysplasia of the hip.

Statistically significant (*P* < .05).

a Statistically significant (*P* < .05).

b OR (95% CI) was obtained from multivariate logistic regression.

As shown in Table 1, COVID-19-positive cases are more likely to be female (97.4% vs 77.2%, *P* = .001), to have visitors at home (sick visit) (89.5% vs 66.7%, *P* = .003), and to stay in the hospital longer (3.2 vs 2.8 days, *P* = .014) than COVID-19-negative cases in the univariate analysis. Other variables (Table 1), such as hospital type (public vs private), city, number of roommates in the hospital, number of doses, and brand of COVID-19 vaccine administered. In multivariate logistic regression analysis, the odds ratio (OR) and 95% confidence interval for females is 12.5 (1.7-91.8, *P* = .013), for having visitors at home is 4.7 (1.6-13.4, *P* = .004), and for staying in the hospital 1 day longer is 1.2 (1.01-1.46, *P* = .043).

Regarding 90-day complications after the surgery, the 2 groups did not show a significant difference (*P* > .05). There was 1 death unrelated to the surgery or COVID-19. In terms of patients' perception of the source of infection, 18 (47.4%, 1.9% of all) of the positive cases believed that they had contracted COVID-19 from the hospital, and the rest (52.6%, 2.1% of all) thought another source was responsible for their COVID-19 infection.

The comparison of vaccinated (October 2021-March 2022) and unvaccinated (April 2020-March 2021) groups is presented in Table 2. The vaccinated group was significantly older than the unvaccinated group (64.1 vs 61.6, *P* < .001), but the other demographics and Charlson Comorbidity Index were similar. Compared to unvaccinated patients, the postoperative COVID-19 rate was not significantly different (3.9% vs 2.4%, *P* = .07). Vaccinated patients who developed postoperative COVID-19 developed insignificantly fewer hospitalizations (10.5 vs 18.2%, *P* = .25) and ICU admissions for COVID-19 (0% vs 16.7%, *P* = .03). Regarding 90-day surgical complications, both groups had comparable rates of VTEs, PJIs/SSIs, and other complications (*P* > .05) (Table 2).

**Table 2:** Comparison of vaccinated and unvaccinated patients who underwent elective joint replacement.

	Two cohorts of arthroplasty patients (N = 1717)		*P* value
	Vaccinated (n = 962, 100%)	Unvaccinated (n = 755, 100%)	
Demographics			
Age (y)	64.1 ± 11.2	61.6 ± 12.5	<.001^a^
Female sex, n (%)	751 (78.1%)	561 (74.2%)	.07
Body mass index (kg/m^2^)	28.2 ± 4.4	28.6 ± 5.1	.08
Charlson Comorbidity Index	0.66 ± 0.96	0.60 ± 0.99	.21
COVID-19-related information			
COVID-19-infection 1 mo after surgery	38 (3.9%)	18 (2.4%)	.07
Mean interval from discharge to COVID-19 (d)	12.7 ± 8.6	10.0 ± 4.2	.12
Hospitalization due to COVID-19	4 (10.5%)	4 (18.2%)	.25
ICU admission due to COVID-19	0	3 (16.7%)	.03^a^
Delayed surgery due to pandemic, n (%)	112 (11.6%)	98 (13%)	.31
Delayed duration (mo)	6.9 ± 6.1	3.7 ± 3.0	<.001^a^
History of COVID-19 infection, n (%)	286 (29.8%)	68 (9.0%)	<.001^a^
History of COVID-19 (months before surgery)	7.3 ± 4.7	4.6 ± 2.6	<.001^a^
Arthroplasty-related complications (3 mo)			
Venous thromboembolism	8 (0.8%)	7 (0.9%)	.83
Periprosthetic joint infection/surgical site infection	11 (1.1%)	10 (1.3%)	.73
Wound complications (dehiscence/ persistent drainage)	61 (6.3%)	32 (4.2%)	.06
Other (periprosthetic fracture, dislocation, joint stiffness)	7 (0.7%)	4 (0.5%)	.61
Revision surgery	0	1 (0.1%)	-
Mortality	1 (0.1%)	0	-

a Statistically significant.

## Discussion

This paper tried to addresses the question of whether we should stop worrying about COVID-19 postoperatively once patients are vaccinated for COVID-19. This study found that 3.9% of fully vaccinated patients (October 2021-March 2022) who underwent a joint replacement surgery developed postoperative symptoms of COVID-19 within 1 month. 4 (10.5%) of them were hospitalized again because of COVID-19 symptoms, and no mortality or ICU admission was detected. In the vaccinated cohort, being female (OR = 12.5), having visitors at home (OR = 4.7), and staying a day longer in the hospital (OR = 1.2) were associated with COVID-19 infection within 1 month after discharge. There was no significant difference in 90-day postoperative complications between COVID-19-positive and COVID-19-negative cases (*P* > .05); although we observed that postoperative COVID-19 did not affect recovery, the low number of cases makes it impossible to draw a conclusion. Both vaccinated and unvaccinated groups of patients had similar 90-day compilations (eg, VTE, SSI/PJI, and wound complications) (*P* > .05).

Concerns exist regarding the effectiveness of vaccines against COVID-19, especially considering emerging VOCs and new virus species [[47], [48], [49], [50], [51]]. Chemaitelly et al. showed that mRNA COVID-19 vaccines' second and booster doses are less than 50% effective against Omicron subvariants and that efficacy decreases to less than 10% afterward [47]. However, it provides a robust and durable defense against COVID-19-related hospitalizations and deaths [47]. The effectiveness of vaccines declined more quickly in people with comorbidities and older adults, and they needed booster shots [48]. Our study did not show a decline in nosocomial COVID-19 infection in the vaccinated group; however, we should pay attention to the country's weekly new cases, which is more, and we had 2 severe peaks in the period of surgery for the vaccinated group. All in all, keeping strict prevention protocols for COVID-19 is imperative. The International Consensus Group recommends that an elective orthopedic surgery be performed only on COVID-19-negative cases, preoperative screening for COVID-19, and a low pathogen transfer rate through COVID-19-free surgical pathways and personal protection equipment and hygiene measures [52]. In the authors' view, these recommendations should continue until the end of the pandemic, even after vaccination.

The CovidSurg Collaborative study at the beginning of the vaccination period found that patients undergoing an elective surgery should be prioritized for vaccination ahead of the general population [53]. It took fewer vaccines (number needed to vaccinate) to prevent 1 COVID-19 death in a year among surgical patients than in the general population, especially for those over 70 years of age [53]. This was a prospective multicenter international cohort study performed on patients who had undergone any surgical procedure, either elective or emergency, routinely carried out by a surgeon in an operating room. Over the course of the study (October 2020-November 2020), participating centers collected data on all consecutive patients in 1 or more predetermined surgical specialties [53]. Vaccination can also reduce postoperative pulmonary complications, resulting in the reduced use of intensive care and overall health care costs [54]. In the absence of any evidence for booster doses, it is reasonable to prioritize these surgical patients for further doses of COVID-19 vaccines. In line with their findings, although our study did not show lower rates of COVID-19 among vaccinated individuals than among nonvaccinated individuals, catastrophic outcomes such as ICU admissions were significantly lower. Therefore, preoperative vaccination could be useful in supporting a safe restart of the elective surgery by reducing the risk of complications related to COVID-19. However, these results were based on only 3 vs 0 patients admitted to the ICU, and hospitalization rates were not significantly different. As a result, the conclusion that vaccines could protect patients from catastrophic events should be taken with caution, as it is not clinically significant. As shown in Figure 3, arthroplasty rates increased following vaccination in our study, and elective joint surgery volumes recovered. Another study by the COVIDSurg Collaborative has found a high rate of 30-day mortality among perioperative COVID-19-positive patients undergoing an elective surgery (18.9%, 53 of 280) [55]. Because of this potential risk, preoperative testing for severe acute respiratory syndrome coronavirus 2 and surgical patient vaccination remain vitally important, especially in areas where the pandemic continues.

Elliott et al. estimated the nosocomial COVID-19 transmission on 14,798 inpatients, defined as an infection per 7 hospital days [56]. COVID-19 transmission during perioperative procedures was significantly linked to the procedural complexity and emergency status. Furthermore, a longer length of stay significantly increased the risk of nosocomial transmission [56]. Similarly, our study revealed that postoperative COVID-19 infections appeared to increase with each extra day of stay in the hospital for arthroplasty (OR = 1.2). Reducing exposure to the hospital setting and decreasing hospitalization days may play a key role in preventing nosocomial COVID-19 infections. Having visitors at home after discharge was also a risk factor for postoperative COVID-19 transmission, highlighting the fact that transmission can occur after discharge and during the recovery period. Relatives and friends who came to see the patients at home might be infected with COVID-19 and put patients at the risk of COVID-19 contraction. Thus, patient home visits by relatives and friends may represent a source of bias that overestimates the COVID-19 rate postoperatively in our study. Surgeons should encourage their patients not to visit public places and to restrict postdischarge home visitors to reduce the risk of COVID-19 postoperatively. Patients should be educated to follow the preventive measures including social distancing and wearing mask if they have visitors at home after the surgery.

In the present study, 2 cities employed 2 different screening protocols: Tehran examined patients twice by RT-PCR before surgery; however, Isfahan took chest x-rays. Postoperative COVID-19 rates did not differ (3.4% vs 4.6%, *P* = .34). According to the study by Mortazavi et al. on 165 arthroplasty patients, only 1 (0.6%) showed postoperative COVID-19 [57]. The authors suggested the preoperative screening protocol, which includes history taking and physical examination, was effective for screening elective surgical patients in a hospital that provides COVID-19 referral services [57]. However, their study includes the first peak of COVID-19, which is not as severe as subsequent peaks. On the other hand, the study by Hamilton et al. including 1000 arthroplasty patients who had PCR tests 48-72 hours before the surgery and were advised to minimize the risk of infection resulted in no case of 30-day postoperative COVID-19 infection [58]. They concluded that preoperative COVID-19 PCR with specific instructions to minimize infection risks along with a COVID-free pathway is safe for patients undergoing arthroplasty [58]. Since some hospitals lack the resources needed to conduct PCR tests routinely and the test has low sensitivity (up to 54% [59]), a screening protocol that includes clinical and radiologic examinations could be adequate, but adding laboratory screening, including the PCR test, may provide more benefits [52,60,61].

Concerns relating to COVID-19 vaccination also exist regarding the risk of VTE, as discussed recently in the international consensus meeting in 2022 on VTE [38]. Their consensus was that COVID-19 vaccination might not increase the risk of VTE and, if it happens, should be treated with an anticoagulant other than heparin. They also noted that patients with a prior history of COVID-19 who undergo orthopedic surgeries are at greater risk of VTE [38]. Our study revealed that vaccinated and unvaccinated patients had similar outcomes regarding complications and VTE, and both had low rates of VTE (0.8% and 0.9%). Based on the results, the authors of this study believe that COVID-19 vaccination may not increase VTE risk in patients undergoing an elective joint replacement with aspirin 325 mg twice daily prophylaxis for 6 weeks. Regarding the prior history of COVID-19 and its impact on postoperative VTE, we had 8 cases complicated with VTE, and none were among the 286 cases with a history of COVID-19 (*P* = .07). Although the population of patients with a previous history of COVID-19 is insufficient to draw definitive conclusions, based on these results, we can say that patients who fully recover from COVID-19 may be good candidates for joint surgeries. Similar to our study, a study by Jungwirth-Weinberger et al. on the impact of previous COVID-19 infection on the complications of elective arthroplasty reported that previous COVID-19 infection did not lead to an extended hospital stay or more complications at the hospital [62]. Furthermore, the same authors found that patients with a positive history of COVID-19 had a low rate of VTE following arthroplasties [63]. Other similar studies revealed the same results [64].

Several limitations were identified in the current study. Due to the low infection rate of COVID-19, it is essential to have a large population to study accurately how infection with COVID-19 may impact the recovery from joint surgeries and complications. Second, patients were monitored and screened for symptoms of COVID-19, and asymptomatic cases were missed. To achieve a more accurate rate, additional studies will be required using both laboratory and imaging screening tools. Additionally, phone calls for following up on patients were subject to limitations because we were not able to check the patients' temperature, perform physical examinations, or evaluate further to determine whether asymptomatic cases existed. Furthermore, we had a loss to follow-up rate of 16%, which may have affected the results highlighting the necessity of a complete follow-up. Finally, various patients in different cities and locations with different vaccination methods can create bias. This study's strength is that it is a multicenter study with a large population and prospective follow-up focusing only on elective joint-replacement surgeries.

## Conclusions

In the light of emerging VOCs and new viruses, there are concerns about the effectiveness of vaccines against COVID-19. In the vaccinated cohort, postoperative COVID-19 incidence was 3.9%, with a 10.5% rehospitalization rate. Although vaccination provides some protection against COVID-19 infection following arthroplasties, it does not guarantee that the patient will not contract the disease, especially in areas where the disease is prevalent. Having visitors at home and prolonged hospital stays are 2 important modifiable factors that patients should minimize for their safety. Both vaccinated and unvaccinated patients had a similar rate of complications and VTEs. It is still important to take precautions, especially when a variant is spreading in the community, even if the patients are vaccinated.

## Acknowledgments

The authors would like to thank Dr. Erfan Sheikhbahaei for introducing the idea and helping in the management of the project. The authors would like to thank Faezeh Niknam for her help in gathering data and for her commitment to the task.

## Conflicts of interest

The authors declare there are no conflicts of interest.

For full disclosure statements refer to https://doi.org/10.1016/j.artd.2022.09.005.

## Appendix A Supplementary data

Conflict of Interest Statement for Moharrami

Conflict of Interest Statement for Ghaderi

Conflict of Interest Statement for Motififard

Conflict of Interest Statement for Salimi

Conflict of Interest Statement for Hosseini-Dolama

Conflict of Interest Statement for Mortazavi

Conflict of Interest Statement for Mirghaderi

Conflict of Interest Statement for Mirghaderi
